# COVID-19 Diagnosed by Real-Time Reverse Transcriptase-Polymerase Chain Reaction in Nasopharyngeal Specimens of Suspected Cases in a Tertiary Care Center: A Descriptive Cross-sectional Study

**DOI:** 10.31729/jnma.5383

**Published:** 2021-05-31

**Authors:** Narayani Maharjan, Niresh Thapa, Bibek Pun Magar, Muna Maharjan, Jiancheng Tu

**Affiliations:** 1 Program & Department of Clinical Laboratory Medicine, Center for Gene Diagnosis, Zhongnan Hospital of Wuhan University, Wuhan, China and Department of Molecular Laboratory, Karnali Academy of Health Sciences, Jumla, Nepal; 2 Department of General Practice and Emergency Medicine, Karnali Academy of Health Sciences, Jumla, Nepal; 3 Department of Clinical Biochemistry, Karnali Academy of Health Sciences, Jumla, Nepal; 4 School of Nursing and Midwifery, Karnali Academy of Health Sciences, Jumla, Nepal; 5 Program & Department of Clinical Laboratory Medicine, Center for Gene Diagnosis, Zhongnan Hospital of Wuhan University, Wuhan, China

**Keywords:** *COVID-19*, *prevalence*, *reverse transcriptase-polymerase chain reaction*, *severe acute respiratory*, *syndrome coronavirus 2*

## Abstract

**Introduction:**

The emergence of severe acute respiratory syndrome coronavirus-2 pandemic is critically challenging the whole world. The real-time reverse transcriptase-polymerase chain reaction is the most widely used confirmatory test for COVID-19 detection. This study aimed to find out the prevalence of COVID-19 infection detected by gold standard reverse transcriptase-polymerase chain reaction test in a tertiary care center of Nepal.

**Methods:**

A descriptive cross-sectional study was conducted in Karnali Academy of Health Sciences from May to August 2020 after taking ethical approval from the Institutional Review Committee of Karnali Academy of Health Sciences, Jumla. Convenient sampling was used. A total of 361 participants enrolled in this study who have done real-time reverse transcriptase-polymerase chain reaction for screening of COVID-19 infection. Also, a designated questionnaire was obtained from persons with a travel history and close contact. Statistical Package for the Social Sciences software was used for the statistical analysis. Point estimate at 95% Confidence Interval was calculated along with frequency and proportion for binary data.

**Results:**

The prevalence of COVID-19 was 167 (46.3%) (95% Confidence Interval= 41.16-51.44) by real-time reverse transcriptase-polymerase chain reaction test. Out of 361 samples, 339 (93.9%) were male and 22 (6%) were female. The highest frequency of the participants belongs to the age groups of 20-40 years.

**Conclusions:**

The findings showed a high prevalence of COVID-19 detected by reverse transcriptase-polymerase chain reaction test. Further studies are necessary to improve the precision of prevalence estimations.

## INTRODUCTION

The COVID-19 is a pandemic disease caused by Coronavirus.^1^ In Nepal, the first case was detected in January 2020 since then there has been an increase in the cases. At the beginning of the COVID-19 pandemic in Nepal, cases were diagnosed mainly by two methods. Rapid diagnostic test detects the presence of antibodies to Severe Acute Respiratory Syndrome causing coronavirus-2 which identifies previous SARS-CoV-2 infection and help to confirm the presence of current infection.^2^ The gold-standard test, real-time reverse transcriptase-polymerase chain reaction detects the presence of SARS-CoV-2 virus in the early phase of infection.^3,4^

The estimation of prevalence is essential for managing and controlling the spread of infection and diseases and for the planning of health care services and epidemiological purposes.^5^

Hence, this study aimed to find out the prevalence of COVID-19 cases detected by real-time RT-PCR test in a tertiary care center of Nepal.

## METHODS

It was a descriptive cross-sectional study, conducted from May to August 2020 in Karnali Academy of Health Sciences (KAHS-Teaching Hospital). This study was approved by the institutional review committee (IRC) of Karnali Academy of Health Sciences, IRC KAHS 077/078/04. There was an increase in the number of COVID-19 suspected cases who came from other countries to Nepal and close contact. Suspected cases for COVID-19 were recruited for the study. The incomplete data were excluded from the study. Also, a designated questionnaire was obtained from persons with a travel history and close contact. Convenient sampling was done and the sample size was calculated using the formula,


$$\begin{array}{ll} \text{n} & \text{= }{\text{Z}}^{\text{2}}\times \text{p}\times q\;/{\text{e}}^{\text{2}}\\ & \text{= }{\left(1.96\right)}^{\text{2}}\times 0.5\times (1-0.5)/{(\text{0.06)}}^{\text{2}}\\ & \text{= }267 \end{array}$$

Where,

n = minimum required sample size
Z = 1.96 at 95% Confidence Interval (CI)
p = prevalence taken as 50% for maximum sample size
q = 1-p
e = margin of error, 6%

The minimum required sample size calculated was 267. The non-response rate was adjusted at 10% of the sample size, the calculated sample size was 294. However, a total of 361 samples were enrolled in this study.

All the data was provided by corresponding health centers where the mass screening for COVID-19 was performed. RT-PCR were done at the time of swab collection for RT-PCR in the respective regions. The nasopharyngeal specimens used in this study had been collected from the suspected participants for COVID-19 diagnosis at different places of Karnali Province, placed in viral transport media (VTM), and sent to the KAHS-Teaching Hospital Laboratory for testing.

Data were retrieved from the medical record book and laboratory record book of KAHS-Teaching Hospital. A standard format was prepared to collect the information such as RT-PCR test result, date, address, age, gender, ethnicity, and travel history.

RT-PCR test was performed in the hospital laboratory as per the WHO guidelines and standard protocol for laboratory testing of COVID-19.^6^

The data were collected and sorted using Microsoft Excel (Microsoft Corporation, New York, USA) software; Statistical Package for the Social Sciences version 16.0 was used for the statistical analysis. Point estimate at 95% Confidence Interval was calculated and descriptive statistics were reported as frequency and percentage.

## RESULTS

**Figure 1.**
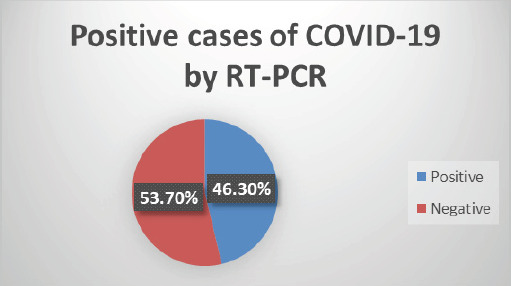
Prevalence of COVID-19 infection determined by RT-PCR.

A total of 361 samples were included in this study. The prevalence of COVID-19 cases detected by RT-PCR was 167 (46.3%) (95% Confidence Interval= 41.16-51.44) (Figure 1).

Out of 361 samples, 339 (93.9%) were male and 22 (6%) were female. The highest frequency of the participants belongs to the age groups of 20-40 years. Dalit and Chhetri ethnic groups occupied the highest frequency of 167 (46.2%) and 109 (30.1%) respectively. Among the participants, 348 (96.3%) have travel history from India. The maximum participants were from Dailekh 300 (83.1%) (Table 1).

**Table 1:** Sociodemographic characteristics.

Variables	n (%)
**Age**	
≤20	111 (30.7)
20-40	208 (57.6)
≥40	42 (11.6)
**Gender**	
Male	339 (93.9)
Female	22 (6.0)
**Ethnicity**	
Janajati	23 (6.3)
Chhetri	109 (30.1)
Brahmin	24 (6.6)
Thakuri	20 (5.5)
Dalit	167 (46.2)
Others	18 (4.9)
**Travel History**	
India	348 (96.3)
Others	8 (2.2)
Not available	5 (1.4)
**District**	
Kalikot	29 (8.0)
Dailekh	300 (83.1)
Bajura	29 (8.0)
Jumla	3 (0.8)

The distribution of different demographic characteristics among COVID-19 positive cases diagnosed by RT-PCR showed the highest frequency of SARS-CoV-2 infection were in Dalit 82(49.1%) followed by Chhetri 44 (26.3%)Brahmin 8 (4.8%). In the case of ethnicity, the highest frequency was found in Dailekh163 (97.6%) followed by Kalikot 4 (2.4%) (Table 2).

**Table 2:** Distribution of demographic characteristics among positive COVID-19 cases diagnosed by RT-PCR (Total positive cases, n = 167).

Characteristics	RT-PCR Positive n (%)	Total n (%)
**Age**		
Under 20	64 (38.3)	111 (30.74)
20-40	91 (54.5)	208 (57.61)
Above 40	12 (7.2)	42 (11.63)
**Gender**		
Male	162 (97.0)	339 (93.90)
Female	5 (3.0)	22 (6.09)
**Ethnicity**		
Janajati	15 (8.9)	23 (6.37)
Chhetri	44 (26.3)	109 (30.19)
Brahmin	8 (4.8)	24 (6.64)
Thakuri	2(1.2)	20 (5.54)
Dalit	82 (49.1)	167 (46.26)
Others	16 (9.6)	18 (4.9)
**District**		
Kalikot	4 (2.4)	29 (8.03)
Dailekh	163 (97.6)	300 (83.10)
Bajura	0 (0)	29 (8.03)
Jumla	0 (0)	3 (0.83)

## DISCUSSION

COVID-19 has become a pandemic in the world. The true burden of SARS-CoV-2 disease in developing countries like Nepal is difficult to estimate due to low resources, low number of testing, and poor documentation.^7^ This cross-sectional study was performed to find out the COVID-19 prevalence diagnosis by the gold standard confirmatory real-time RT-PCR test. Based on the available data, we estimated the prevalence of SARS-CoV-2 infection. Among 361 samples, the prevalence of COVID-19 infection was 167 (46.3%). In a study carried out among healthcare workers in Brazil, a high prevalence (42.37%) of SARS-CoV-2 infection was found.^8^ But another study by Lahner E, et al.^9^ showed the prevalence of only 2.7% which was very low as compared with the present study result.

In the early phase of the pandemic, RDT was performed for screening of COVID-19 infection in the mass of people with travel history. But there is very limited evidence of clinical sensitivity for commercially available kits for the diagnosis of COVID-19. The WHO also does not currently recommend the use of rapid diagnostic tests for patient care. Though, conducting research and finding its possible utility is highly encouraged.^10^ Some published studies have shown that rapid tests based on host antibodies (IgM/ IgG), are non-specific for COVID-19, and may have very low sensitivity and specificity.^11,13^

There were predominantly high male (94%) participants and most of them were in the productive age group. The study results showed a high prevalence of SARS-CoV-2 infection among the asymptomatic general population of the western region of Nepal. This showed that the higher rate of spread of the disease in the population with travel history. Most of the participants (96.3%) had a travel history from India. The majority of participants who migrated to India for employment were from the Dailekh district and the ethnic group of Dalits was affected mostly. This is because the socioeconomic development of the Karnali region is poor and Human Development Index (HDI) is below 0.43^14^ so that mostly males from poor family especially Dalit migrate to India for employment.

However, there were some limitations of the study such as the prevalence calculated maybe being an underestimation as the individual number tested is low so, the study could not generalize the whole population. The estimated prevalence is only a consideration of those who have tested rather than the true prevalence at the whole population level. Also, the included participants were asymptomatic and the available information was limited so that it was not possible to include different variables for comparison.

## CONCLUSIONS

The findings showed a high prevalence of COVID-19 diagnosed by RT-PCR test. Further population-based studies are necessary to improve the precision of prevalence estimations. Also, high awareness concerning the spread and transmission of SARS-CoV-2 infection is required among general people.
